# Surface Modification of Polyethylene Terephthalate Track-Etched Membranes by 2,2,3,3,4,4,5,5,6,6,7,7-Dodecafluoroheptyl Acrylate for Application in Water Desalination by Direct Contact Membrane Distillation

**DOI:** 10.3390/membranes14070145

**Published:** 2024-06-25

**Authors:** Aigerim Kh. Shakayeva, Arman B. Yeszhanov, Alexander N. Borissenko, Murat T. Kassymzhanov, Ainash T. Zhumazhanova, Nikolai A. Khlebnikov, A. K. Nurkassimov, Maxim V. Zdorovets, Olgun Güven, Ilya V. Korolkov

**Affiliations:** 1 The Institute of Nuclear Physics, Ibragimov Str., 1, Almaty 050032, Kazakhstan; shakayevaa19@gmail.com (A.K.S.); a.yeszhanov@inp.kz (A.B.Y.);; 2 JSC “Park of Nuclear Technologies”, Kurchatova Str. 18/1, Kurchatov 071100, Kazakhstan; 3 Ural Federal University, Mira Str. 19, Yekaterinburg 620002, Russia; na.khlebnikov@urfu.ru; 4 Department of Chemistry, Hacettepe University, Ankara 06800, Turkey; guven@hacettepe.edu.tr

**Keywords:** track-etched membranes, membrane distillation, photoinitiated graft polymerization, hydrophobic membrane, poly(ethylene terephthalate)

## Abstract

In this work, the surfaces of poly (ethylene terephthalate) track-etched membranes (PET TeMs) with pore sizes of 670–1310 nm were hydrophobized with 2,2,3,3,4,4,5,5,6,6,7,7-dodecafluoroheptyl acrylate (DFHA) by photoinitiated graft polymerization. Attenuated total reflection FTIR spectroscopy (ATR-FTIR), scanning electron microscopy (SEM) coupled to an energy-dispersive X-ray spectrometer (EDX), and contact angle measurements were used to identify and characterize the TeMs. The optimal parameters for graft polymerization were determined as follows: polymerization time of 60 min, monomer concentration of 30%, and distance from the UV source of 7 cm. The water contact angle of the modified membranes reached 97°, which is 51° for pristine membranes. The modified membranes were tested for water desalination using direct contact membrane distillation (DCMD) method. The effects of membrane pore size, the degree of grafting, and salt concentration on the performance of membrane distillation process were investigated. According to the results obtained, it has been concluded that large pore size hydrophobic TeMs modified by using DFHA could be used for desalinating water.

## 1. Introduction

Water is an essential resource for the survival and growth of life, as well as for sustaining the environment. However, the vast majority of water on Earth is too salty for human use. The issue of access to fresh water is one of the most serious problems of modern times, affecting many aspects of life on Earth [1]. This problem is enhanced by the continuous growth of the population, increasing urbanization, the deterioration of water quality due to pollution from industry and agriculture, and the risk of droughts resulting from climate change. Addressing the issue of fresh water requires a comprehensive, holistic approach. This includes the effective management of water resources, improving infrastructure for water purification and distribution, and implementing innovative technologies for water desalination [2].

Seawater desalination has expanded rapidly over the past few decades, mainly to provide water for municipal and industrial use in water-scarce regions. Several desalination methods are being researched to improve efficiency and cost [3]. Promising techniques include reverse osmosis, electrodialysis, thermal distillation, nanofiltration, and membrane distillation (MD) [4,5]. Among all these technologies, MD has a lot of advantages, including operation at low temperatures and hydrostatic pressures, high levels of water recovery, high salt rejection rates (especially for samples with a salinity between 70 and 300 g salt per kg solution), and less sensitivity to membrane fouling [6]. MD is a promising technology for desalination, wastewater treatment, and the purification of various liquids due to its energy efficiency and versatility. MD is widely used in seawater and groundwater desalination, wastewater and natural water treatment to remove heavy metals, liquid radioactive wastes, and pesticides [7,8,9]. This is due to its driving force, which is the vapor partial pressure difference across the membrane, and its ability to be powered by solar thermal energy, geothermal energy, or waste energy [10,11]. MD can be realized in various arrangements, including direct contact membrane distillation (DCMD), vacuum membrane distillation (VMD), air gap membrane distillation (AGMD) and sweeping gas membrane distillation (SGMD), and their modifications [12,13,14].

Recently, there has been a growing interest in enhancing the performance and durability of MD membranes to meet the increasing demands of industrial applications. MD is a thermally driven process that is influenced by the structure of the membrane. Membranes for MD are required to have main characteristics including porosity, hydrophobicity, high liquid entry pressure (LEP), high permeability, low fouling rate, low thermal conductivity, excellent chemical and thermal stability and mechanical strengths [15]. Polymeric, inorganic, and ceramic membranes have gained significant attention as materials highly suitable for MD applications. Polymeric membranes are widely used in MD process as a cheaper alternative to ceramic and inorganic membranes. The polymeric membranes most commonly used are poly(vinylidene fluoride) (PVDF), polyethylene (PE), polypropylene (PP), polytetrafluoroethylene (PTFE), polydimethylsiloxane (PDMS), and poly(ethylene terephthalate) (PET) [16]. MD membranes can be produced using various methods, such as track etching, stretching, phase inversion, electrospinning, and combinations of different methods [17,18]. 

Track-etched membranes (TeMs) are a highly versatile and extensively researched material used for water filtration, cell cultivation, catalysts, sensors, and other applications [19,20,21]. TeMs are manufactured by irradiation of thin polymer films with heavy ions to create distinct tracks and subsequent chemical etching. Chemical etching creates cylindrical pores along the tracks by controlling the concentration and temperature of etching solution. This enables full traceability of the membrane properties. TeMs are suitable for use in MD processes due to their narrow pore size distribution, small thickness, flexibility, and chemical and biological compatibility [22]. Poly(ethylene terephthalate) (PET) membranes have emerged as a viable candidate for MD due to their mechanical strength, thermal stability, and chemical and heat resistance [23]. However, the hydrophilic properties of PET are a major disadvantage when it comes to MD applications. Optimizing their surface properties to achieve a high level of hydrophobicity is critical in the development of PET membranes for MD [24]. This is essential to facilitate the vapor phase transport of water molecules while preventing the passage of liquid water and other contaminants through the pores. To address this challenge, researchers have explored various surface modification techniques, including the incorporation of fluorine-containing compounds [25]. 

Fluorine-containing compounds are well-known for their exceptional hydrophobic properties. Fluorine (F) atoms have a high electronegativity, low polarizability, and a small van der Waals radius (1.32 Å). This allows the formation of strong C-F bonds, giving polymers good thermal and chemical stability and a low surface energy. The high bond dissociation energy of 485 kJ/mol is responsible for the unique properties of the C-F bond. The stability of a compound increases with the number of C-F bonds [26]. A recent work [27] describes a method for modifying ceramic membranes with perfluoroalkylsilanes of different fluorine alkyl chain lengths by graft polymerization. The grafting efficiency in terms of surface coverage has been studied by determination of the contact angle with water and glycerol. The results show an increase in contact angle (from 126° to 136°) with increasing fluoroalkyl chain length. Zhang W. et al. [28] modified the PVDF membrane by immobilizing silica nanoparticles, followed by functionalization with polydopamine (PDA) and fluorosilanization with the silane coupling agent 1H, 1H, 2H, 2H-perfluorooctyltrichlorosilane. The resulting superhydrophobic and oleophobic membrane was successfully used in a DCMD process using a mixed solution of inorganic salts, organic matter, and surfactant as the feed. In our paper [29] we showed that the surface of PET TeMs modified by using dichlorodimethylsilane and 1H, 1H, 2H, 2H-perfluorododecyltrichlorosilane increased the wettability edge angle to 134°. The membrane that had been modified was subsequently employed in DCMD for water desalination, achieving an efficiency of 99.5%. The efficiency and reliability of membrane distillation processes was improved by providing a robust hydrophobic barrier that effectively rejects liquid water while allowing water vapor to pass.

Moreover, in our previous study [30], PET TeMs with pore diameters ranging from 724 to 1305 nm were modified with lauryl methacrylate by photoinitiated graft polymerization. Modified membranes were successfully used for water desalination by DCMD. Additionally, hydrophobic PET TeMs with large pore sizes were prepared by photoinitiated graft polymerization of 2,2,3,3,4,4,5,5,6,6,7,7-dodecafluoroheptyl acrylate (DFHA). The modified PET TeMs were applied for desalinating water with salt concentration ranging from 7.5 to 30 g/L using the DCMD method. 

## 2. Materials and Methods

### 2.1. Chemicals

2,2,3,3,4,4,5,5,6,6,7,7-dodecafluoroheptyl acrylate (95%, Sigma-Aldrich, Tokyo, Japan) (DFHA), N,N—dimethylformamide (99.9%, Sigma-Aldrich, Darmstadt, Germany) (DMF), benzophenone (BP) (97%, Sigma-Aldrich, Darmstadt, Germany), ethanol (98%, Sigma-Aldrich, France, St. Quentin Fallavier), and 2-propanol (99.8%, Sigma-Aldrich, France, St. Quentin Fallavier). The monomer was passed through an aluminum oxide chromatographic column to remove the stabilizers. Deionized water (18.2 MΩ) was used for the preparation of all solutions.

### 2.2. Method of Producing and Modification of PET TeMs

TeMs were created using DC-60 ion acceleration (Astana branch of the Institute of Nuclear Physics of the Republic of Kazakhstan). PET TeMs were obtained from Hostaphan^®^ brand PET films (Mitsubishi polyester film, Wiesbaden, Germany) with a thickness of 12 μm and irradiated by high-energy Kr ions at 1.75 MeV/nucleon. The surface density of pores was constant at *n* = 1 × 10^6^ ions/cm^2^. Pore systems were formed by parallel, unconnected channels at right angles to the TeMs’ surfaces, the pore diameter *d* was varied. The variation of the pore size was achieved by chemical etching time. Track-etching of the irradiated PET films was performed in a 2.2 M NaOH solution at 85 °C. Then, the membranes were washed in acetic acid and deionized water and air-dried at room temperature. Before the graft polymerization process, an oxidation process in hydrogen peroxide (0.3 M, pH = 3 (HCl)) under UV-light for 180 min on each side was carried out in order to increase the concentration of benzophenone to be immobilized on the PET surface. After oxidation PET TeMs were washed twice in deionized water and dried in air at room temperature [31]. 

PET TeMs’ surfaces were modified by photo-induced graft polymerization. The membranes were immersed in 5% initiator (BP) in DMF for 24 h. The adsorbed BP concentration was 580 μmol/g. The concentration was determined by the method described in [32]. After 24 h, the samples were washed with ethanol and dried in air. The monomer concentration ranged from 10–30%, and 2-propanol was chosen as the solvent. Before graft polymerization, the reaction mixture was flushed with Ar to remove the dissolved oxygen. During irradiation the reaction vessel was covered with a thin poly(vinyl chloride) (PVC) film. Graft polymerization was carried out using OSRAM Ultra Vitalux E27 lamp (Munich, Germany) (UVA: 315–400 nm, 13.6 W; UVB: 280–315 nm, 3.0 W) for 30–60 min, and TeMs were placed 7 cm from the UV lamp. Irradiated films removed from reaction vessel were washed in 2-propanol and water, and air-dried.

### 2.3. Characterization Technics

The functional group measurement was executed with a FT-IR spectrometer InfraLUM FT-08 (Saint-Petersburg, Russia) with an attenuated total reflection (ATR) attachment (GradiATR, PIKE, USA) (range 400–4000 cm^−1^, 20 scans at resolution 2 cm^−1^).

The morphology and elemental analysis of PET TeMs were obtained with a Hitachi TM 3030 (Tokyo, Japan) with a Bruker XFlash MIN SVE EDX instrument (Massachusetts, USA) at 15 kV acceleration voltage. The EDX spectrum is selected in 120 s. Prior to analysis, the sample is coated with a layer of gold. The analyzed results are presented as averages based on three data points. The average diameter value of the pores was measured.

The water contact angle (CA) was measured using a Digital Microscope with 1000× magnification (Suzhou, China) at room temperature. The CA was evaluated using the static drop method. The measurement was consecutively repeated five times at the same position. The CA was found using ImageJ1.4.3.67 programs with Drop Analysis-Drop Snake plugins.

The surface area of the PET TeMs was measured based in N_2_ adsorption isotherms using V-Sorb 2800P BET surface area and porosity analyzer (Gold App Instruments corp., Xi’an, China).

### 2.4. Membrane Distillation Tests

Membrane distillation tests were performed using a DCMD, which consisted of an MD cell, a circulating flow of feed water and permeate, digital balance, conductometer, data acquisition system, and four Type-T thermocouples. A schematic diagram of DCMD was shown in our previous work [33]. The tested membrane was mounted in the DCMD cell (0.127 × 0.9 m), between the feed flow channel and condensing surface. The effective area of PET TeMs was 0.72 m^2^. Permeate flow rate (227 ± 3 mL/min) and feed flow rate (453 ± 3 mL/min) were controlled using Easy load Cole-Parmer Masterflex L/s 77200-62 (Cole-Parmer Instrument Co., Vernon Hills, IL, USA). The temperature of feed was 80 °C and the temperature of permeate was 10 °C (∆T = 70 °C). The weight (± 0.01 g) of collected distilled water was continuously recorded (every 30 s) using a digital balance to determine the flow rate of the tested membranes. To calculate the salt rejection during membrane distillation, the conductivity of the feed and distilled water was measured with a Hanna Instruments Conductometer HI2030-01 (HANNA Instruments, Cluj, Romania). Thermocouples were used to measure the liquid temperature at the inlet and outlet of the permeate flow channel and the feed flow channel, respectively. The data collected during MD experiments were transferred to the computer and monitored in real-time. The water flux (*W*) was calculated using Equation (1):

(1) $$W=\frac{∆m}{A∆t}$$

where:

*W*—water flux, g/m^2^·h;
∆*m*—mass difference in permeate side, g;
∆*t*—time of MD process, h;
*A*—effective area of membrane, m^2^.

Degree of salt rejection (*R*) was calculated by the formula:

(2) $$R=100-\left(\frac{C_{r}}{C_{f}}\cdot 100\%\right)$$

(3) $$C_{r}=\frac{∆\sigma \cdot 1000}{2.3}$$

(4) $$C_{fic}=\frac{∆m\cdot C_{feed}}{m_{p}}$$

where:

R—degree of salt rejection, %;
$C_{r}$—the concentration of NaCl in permeate side after MD, g/L;
$C_{f}$—the theoretical concentration of NaCl, g/L;
∆σ—difference in conductivity of permeate solution before and after MD, μS/cm;
2300 μS/cm—the change in the conductivity of the solution with the addition of 1 g/L of NaCl;
∆m—the permeate gain after MD, g;
$C_{feed}$—the initial concentration of salt in feed solution, g/L;
$m_{p}$—the mass of water from the permeate side before MD, g.

## 3. Results and Discussion

### 3.1. Fabrication of Hydrophobic PET TeMs

**Figure 1:**
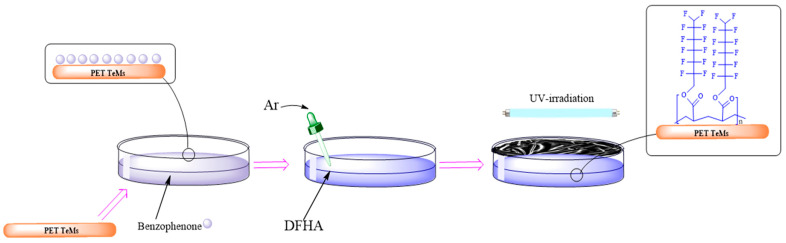
Scheme of graft polymerization of DFHA.

Graft polymerization is a useful method for introducing a range of functional groups onto the surface of a polymer. Introducing new reactive sites can modify polymeric surface morphologies, enhancing specific material properties [34]. Modification of PET TeMs surfaces was carried out by photo-induced graft polymerization of DFHA to create a hydrophobic layer. Figure 1 shows the scheme of the photoinitiated graft polymerization of DFHA. The hydrolysis and subsequent oxidation of PET occurred after chemical etching produced carboxyl and hydroxyl groups on the chains. These groups are involved in the process of attaching the monomers in the graft polymerization process [35]. 

The graft polymerization was affected by distance from the UV lamp, reaction time, and monomer concentration. Chemical grafting reactions involve the use of an initiator. BP is a photoactive compound that is commonly used to functionalize various material surfaces, such as plastics. PET TeMs were immersed in BP solution, for adsorption at the membrane surface. When exposed to UV light, BP undergoes a photoreaction with the abstraction of H and the formation of radicals that will attack the membrane surface. Oxidation of PET TeMs leads to adsorption of a higher concentration of benzophenone onto the surface of PET TeMs and the subsequent concentration of radicals [32,36,37].

The effect of the distance from the UV source on the polymerization process has been studied. The optimum distance was found to be 7 cm. Increasing the distance up to 10 cm showed poor polymerization as no characteristic peaks of DFHA were observed in the FTIR spectra. Conversely, decreasing the distance to 5 cm showed a large amount of homopolymer formation on the membrane surface, with the pores being closed by the homopolymer. This is due to the rapid evaporation of the solvent from the reaction mixture. A reduction in the distance between the lamp and the reaction mixture from 10 cm to 5 cm resulted in a significant increase in the temperature of the mixture, which rose from 38 °C to 83 °C. Consequently, at a distance of 5 cm, the boiling point of 2-propanol was observed.

**Figure 2:**
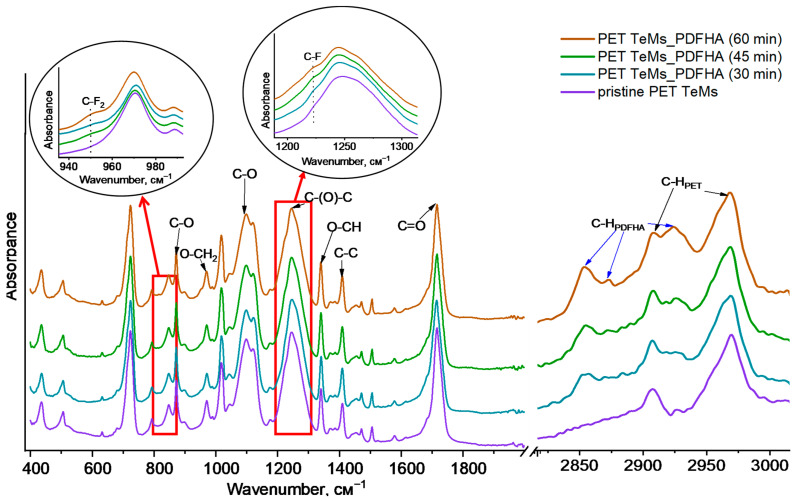
FTIR spectra of pristine and modified PET TeMs with various grafting time.

The chemical structures of the initial and modified PET TeMs with different grafting time were evaluated using FTIR spectroscopy as shown in Figure 2. In the FTIR spectra, absorption peaks of the initial PET TeMs were observed at 2970 cm^−1^ (aromatic C-H), 2911 cm^−1^ (aliphatic C-H), 1712 cm^−1^ (C=O), 1471 cm^−1^ (CH_2_ bending), 1411 cm^−1^ (CH bending), 1342 cm^−1^ (CH_2_ wagging), 1244 cm^−1^ (vibrations of C(O)-O bonds), 1019 cm^−1^ (in plane vibration of benzene), 970 cm^−1^ (O-CH_2_ stretching), and 847 cm^−1^ (C-C stretching) [38]. The grafting of DFHA onto the membrane surface gave rise to the appearance of new peaks in the FTIR spectra related to the DFHA structure, such as those at 2925 cm^−1^ and 2855 cm^−1^ (C-H aliphatic), 950 cm^−1^ (C-F_2_ bending), and 1221 cm^−1^ (C-F bending) [39]. These characteristic peaks were also observed in the FTIR spectra with different concentrations of monomers (Figure S1).

Figure 2 shows that peak intensities increased with increasing graft polymerization time and monomer concentration. To quantitatively evaluate this, absorbance values for C-F_2_ groups at A_950_/A_1410_ were calculated and are presented in Table 1 for different parameters.

**Figure 3:**
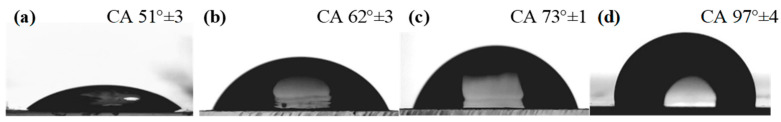
CAs of original PET TeMs (**a**), and grafted PET TeMs for 30 min (**b**), 45 min, (**c**) and 60 min (**d**).

The wettability of the original and modified PET TeMs surfaces with different grafting time and DHFA concentrations are shown in Figure 3 and Figure S3. The CA of 51° ± 3 that was obtained for the original PET TeMs shows the hydrophilic nature of the surface. After graft polymerization, it was observed that the CA increased progressively from 62° ± 3 to 97° ± 4. The highest wettability was obtained at a monomer concentration of 30% and a reaction time of 60 min, with a mean value of 97° ± 4°. The water drop remained compact and did not spread over a long period of time. The water droplet spread faster and penetrated the membrane as the monomer concentration decreased [40]. 

**Figure 4:**
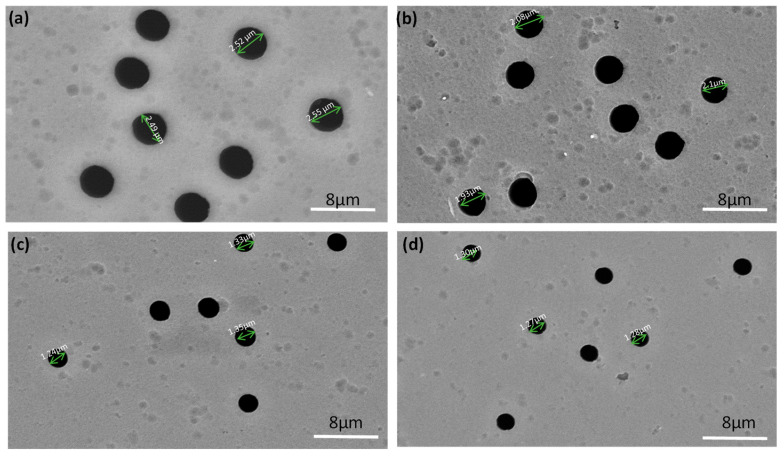
Morphologies of pristine PET TeMs surface (**a**) and those modified with 30% monomer concentration and at different grafting times: 30 min (**b**), 45 min (**c**) and 60 min (**d**).

The morphological differences between the original and modified PET TeMs were observed using SEM. Results are presented in Figure 4 and Figure S3. The SEM images show a smooth surface and a slight reduction in the pore dimensions of the TeMs, indicating the formation of a polymer layer.

The pores were not completely blocked by the polymer due to the initial large diameter of the TeMs pores and by keeping the grafting levels low. According to the data presented in Table 2, it can be observed that the pore diameter after grafting tends to decrease with increasing graft polymerization time and monomer concentration. A slight decrease in pore diameter is observed at the lowest grafting time (30 min) and lowest monomer concentration (10%). The formation of a homopolymer, which closes the membrane pores, occurs with increasing reaction time (more than >60 min) and monomer concentration (more than >30%).

The burst strength was determined using a pressure that would damage a circular sample with a surface area of 1 cm^2^. The original PET TeMs had a burst strength of more than >0.449 MPa. For grafted membranes with pore diameters of 670 nm, the burst strength was 0.380 MPa, for diameters of 890 nm it was 0.404 MPa, and for diameters of 1310 nm it was 0.412 MPa (0.449 MPa—the maximum pressure that can be applied by the equipment). The BET surface area of initial PET TeMs was 1.726 m^2^/g. The pristine PET TeMs thickness was 10.5 ± 2 μm. After modification membrane thickness increased and it was 13.2 ± 2 μm. 

The elemental compositions of the PET TeMs surface were studied by EDX. According to the EDX analysis, an increase in the polymerization time and the DFHA concentration resulted in an increase in the fluorine concentration. The highest fluorine concentration was observed in the membrane with the highest monomer concentration (30%) and the longest reaction time (60 min). 

A collective evaluation of the above results shows that the optimal parameters for graft polymerization of DFHA are a 30% monomer concentration with a graft reaction time of 60 min. The results obtained from the EDX and FTIR spectroscopy, SEM images, and CA prove the formation of a polymer layer on the surface of the PET TeMs.

### 3.2. Desalination by DCMD 

**Figure 5:**
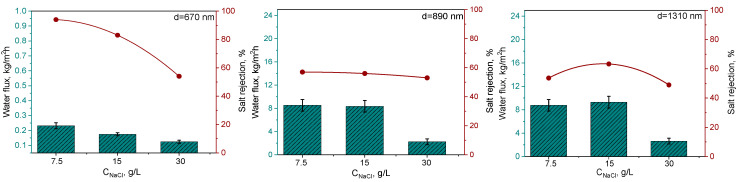
Effect of pore size and grafting degree on water flux and degree of salt rejection at three different concentrations of NaCl.

The desalination process with PET TeMs modified using the optimal grafted conditions was assessed using two feed solutions (7.5, 15 and 30 g/L NaCl in water). Samples of PET TeMs with different pore sizes were utilized in the process. Figure 5 illustrates the salt rejection and water flux of the membranes with different pore diameters and graft levels. After each experiment the membranes were washed in warm water for 12 h to remove any salt remaining on the surface. 

Initially modified membranes with an average pore diameter of 2510 nm were tested for water desalination. However, these membranes did not remove salt from the water. Therefore, the pore diameter of the membranes used was reduced. 

Figure 5 shows that the degree of salt rejection decreased from 94% to 44% with an increase in pore size from 670 nm to 1310 nm. Increasing the pore diameter from 670 to 1310 nm leads to an increase in water flux, as a greater amount of water can pass through larger pores. According to the results obtained, there is a slight decrease in the purification rate with increasing salt concentration. Most ocean water contains about 35 g of salt per liter of water [41]. As shown in Figure 5, increasing the salt concentration from 7.5 to 30 g/L results in a decrease in the water flux from 0.232 to 0.125 kg/m^2^ h for the 670 nm pore size membrane. Increasing the NaCl concentration reduces water activity and the water vapor pressure and increases the water viscosity and the temperature polarization at the membrane surface [42]. However, it should be noted that at pore diameters of 890 and 1310 nm, there is no significant change in performance with increasing salt concentration, as seen for membranes with a pore diameter of 670 nm. For membranes with pore diameters of 890 and 1310 nm, a decrease in performance is observed at a salt concentration of 30 g/L. A high salt concentration increased the risk of membrane fouling, reducing its water flux. As the concentration of NaCl in a solution increases, the viscosity of the solution also increases. A higher viscosity can impede the flow of the solution through the system, thereby reducing the mass transfer rate and consequently the water flux through the membrane. Therefore, the data obtained for salt rejection can be considered competitive if compared with many works reported in the literature and are summarized in Table 3.

## 4. Conclusions

The article presents the results of obtaining hydrophobic track-etched PET membranes with pore sizes of 670–1310 nm through the photoinitiated graft polymerization of DFHA for water purification by membrane distillation. FTIR spectroscopy, and theSEM and CA methods were used to demonstrate the success of PET TeMs surface modification and to determine the optimal conditions for the graft polymerization process. Membranes with a contact angle of 97° were tested for water desalination at NaCl concentrations ranging from 7.5 to 30 g/L, which showed a water desalination rate of 94%.

## Figures and Tables

**Table 1:** Results of area under the peak at 950 cm^−1^ determined from normalized spectra at 1410 cm^−1^ at different grafting times and monomer concentrationd.

№ Sample	Polymerization Time, min	Monomer Concentration, %	Absorbance Value (A_950_/A_1410_)
1	30	30	0.495 ± 0.042
2	45	30	0.539 ± 0.024
3	60	30	0.572 ± 0.014
4	60	20	0.519 ± 0.064
5	60	10	0.491 ± 0.002

**Table 2:** Results of elemental analysis, contact angle, and pore size for PET TeMs with different graft polymerization parameters.

№ Sample	Polymerization Time, min	DFHA Concentration, %	CA, ° ±5°	Pore Size (From SEM Analysis), nm	Concentration of F, %
1	0	-	51	2510 ± 560	-
2	60	10	68	2420 ± 683	0.42 ± 0.2
3	60	20	69	2175 ± 124	0.91 ± 0.5
4	60	30	97	1295 ± 640	0.94 ± 0.48
5	45	30	73	1333 ± 133	0.86 ± 0.15
6	30	30	62	2091 ± 136	0.6 ± 0.4

**Table 3:** An overview of the performance of different membranes in DCMD.

Membrane Material	CA (°)	DCMD Conditions	R, %	Ref.
DHPVC-graft-PEA	95.48 ± 0.79	NaCl 35, 70 and 100 g/L	99.9	[43]
PVDF-co-HFP with F-g-FeOOH	132 ± 1.6	NaCl 10,000 ppm	99.9	[44]
PPO/PS	-	NaCl 35,000 ppm	99.9	[45]
LMP ECTFE	116 ± 2	NaCl 0.6 M	94.95–99.8	[46]
SAN electrospun nanofibers	139.8 ± 0.8	NaCl 35 and 70 g/L	99.9	[47]
PVDF@FGi particles	105–140	NaCl 1 M	99.8	[48]
PES	~102	NaCl 35 g/L	69.8	[49]
SPES@MWCNTs	145	NaCl 3.5%	99.8	[50]
Pure SPES	70	NaCl 3.5%	95.2	
PET TeMs-graft-LMA (700–1300 nm)	94 ± 4	NaCl 7.5, 15 and 30 g/L	91.4	[30]
PET TeMs-graft-DFHA (700–1300 nm)	97 ± 4	NaCl 7.5, 15 and 30 g/L	94	This work

## Data Availability

The data presented in this study are available on request from the corresponding author.

## Supplementary Materials

The following supporting information can be downloaded at: https://www.mdpi.com/article/10.3390/membranes14070145/s1, Figure S1: FTIR spectra of initial and grafted membranes at different monomer concentration; Figure S2: Water contact angle for initial (a) and modified PET TeMs with 10% DFHA (b), 20% DFHA (c) and 30% DFHA (d); Figure S3: SEM images PET TeMs before (a) and after 60 min grafting The monomer concentration: 10% (b), 20% (c) and 30% (d).
