# Supplementary material for: Surface Modification of Polyethylene Terephthalate Track-Etched Membranes by 2,2,3,3,4,4,5,5,6,6,7,7-Dodecafluoroheptyl Acrylate for Application in Water Desalination by Direct Contact Membrane Distillation
Source: Membranes (Basel). 2024 Jun 25;14(7):145. doi: 10.3390/membranes14070145 (PMC11278615; doi:10.3390/membranes14070145)
Supplement: Supplementary file 1 [file membranes-14-00145-s001.zip › membranes-3057385-supplementary.pdf]

Supplementary Materials

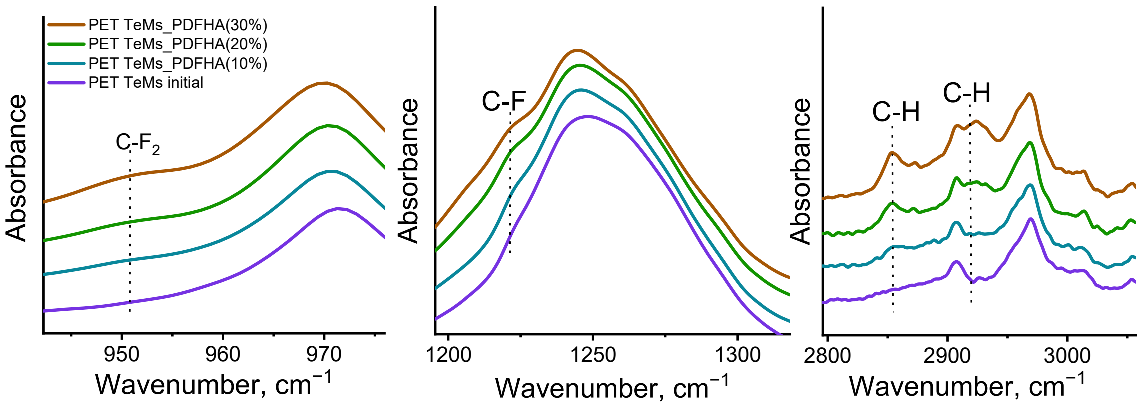

Figure S1. FTIR spectra of initial and grafted membranes at different monomer concentration.

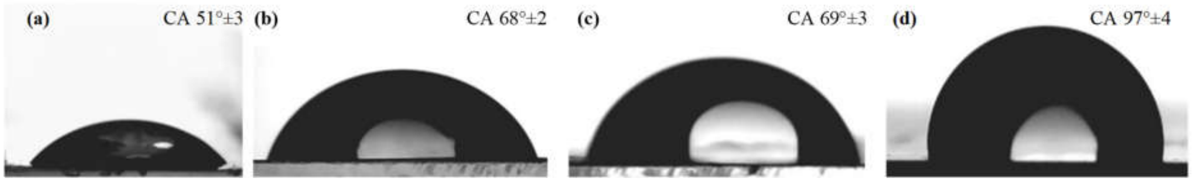

Figure S2. Water contact angle for initial (a) and modified PET TeMs with 10% DFHA (b), 20% DFHA (c) and 30% DFHA (d)

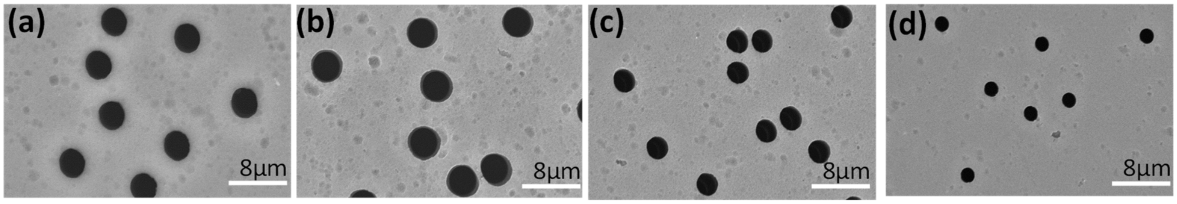

Figure S3. SEM images PET TeMs before (a) and after 60 min grafting The monomer concentration: 10% (b), 20% (c) and 30% (d)
